# Novel Case of Primary Psoas Abscess With No Associated Risk Factors

**DOI:** 10.7759/cureus.82203

**Published:** 2025-04-13

**Authors:** Petar Martinovski, Anthony Bally, Jake Vinton, Sarkis Kouyoumjian

**Affiliations:** 1 Emergency Medicine, Wayne State University School of Medicine, Detroit, USA; 2 Emergency Medicine, Wayne State University Detroit Medical Center, Detroit, USA

**Keywords:** iliopsoas, medical education, primary psoas abscess, psoas abscess, secondary psoas abscess

## Abstract

Psoas abscess is an uncommon and often difficult condition to diagnose due to its vague and variable symptoms. While certain signs such as fever, back pain, and difficulty walking may be associated with it, they are not consistently present in all cases. These abscesses can arise spontaneously or as a result of an underlying condition. Spontaneous cases are more frequently observed in individuals with compromised immune systems or other chronic health issues, although they may occasionally occur without any clear predisposing factors. We describe a case involving a 36-year-old male without known underlying conditions who developed a spontaneous psoas abscess. This case highlights the importance of maintaining a broad diagnostic perspective, even when conventional risk factors are absent, and raises the possibility that other, less well-defined contributors to this condition may exist.

## Introduction

The psoas muscle is a skeletal muscle with an origin at the T12-L5 vertebrae and an insertion at the lesser trochanter of the femur [1,2]. The psoas muscle joins with the iliacus muscle before its final attachment, so both are commonly referred to as the iliopsoas. This muscle serves as one of the primary flexors of the hip and is innervated by the L2, L3, and L4 nerve roots [2-4]. A psoas abscess is a collection of pus within the psoas muscle and commonly yields *Staphylococcus aureus* on fluid culture [5-7]. The classic presentation of a psoas abscess consists of fever, back pain, and an antalgic gait with limited hip range of motion. However, this triad is only present about 30% of the time [1,2,4]. As a result, this is a challenging condition to diagnose due to variable presentations associated with many other conditions. Some conditions with similar presentations of this triad include pyelonephritis, septic arthritis of the hip, and osteomyelitis. Nonspecific symptoms can include vague abdominal pain, malaise, pain that radiates to the hip and thigh, nausea, and weight loss. Therefore, it is paramount to maintain a high degree of clinical suspicion with such a condition. The gold standard for diagnosis is a CT of the abdomen/pelvis. Ultrasound may also be used; however, it is only diagnostic in 41% of the cases and is operator dependent [8]. Management typically involves the use of appropriate antibiotics tailored to the organism grown and drainage of the abscess. A psoas abscess may be defined as primary or secondary, depending on whether there is an associated condition. Primary psoas abscess risk factors include diabetes mellitus, intravenous drug use, AIDS, renal failure, and immunosuppression. In contrast, secondary causes of psoas abscess are due to diseases of the gastrointestinal, genitourinary, musculoskeletal, and vascular systems [3,6,7]. Examples of conditions that can cause a secondary psoas abscess include Crohn's disease, pyelonephritis, osteomyelitis, and infective endocarditis [3,6,7].

## Case presentation

We present the case of a 36-year-old male diagnosed with a primary psoas abscess who presented for evaluation of back pain but lacked all major associated risk factors for this condition. The patient has no past medical history and reported two days of left-sided middle back pain that began while at work. He denied any previous episodes of this pain. He stated that his job involves regularly lifting heavy objects (car parts). The patient described the pain as severe and exacerbated by any movement of the back. It had since spread to the contralateral side and was progressively worsening. Aspirin (acetylsalicylic acid) had failed to relieve his symptoms.

A review of systems was otherwise unremarkable, with no gastrointestinal, genitourinary, neurologic, cardiopulmonary, or other systemic complaints. Additionally, both the patient’s medical and psychosocial histories were unremarkable and noncontributory. Upon presentation to the ED, the patient was hypertensive and tachypneic. Laboratory results were within normal limits except for a mild leukocytosis of 12.9 (10^9/L) with a left shift. The patient appeared to be in significant pain and distress.

**Figure 1:**
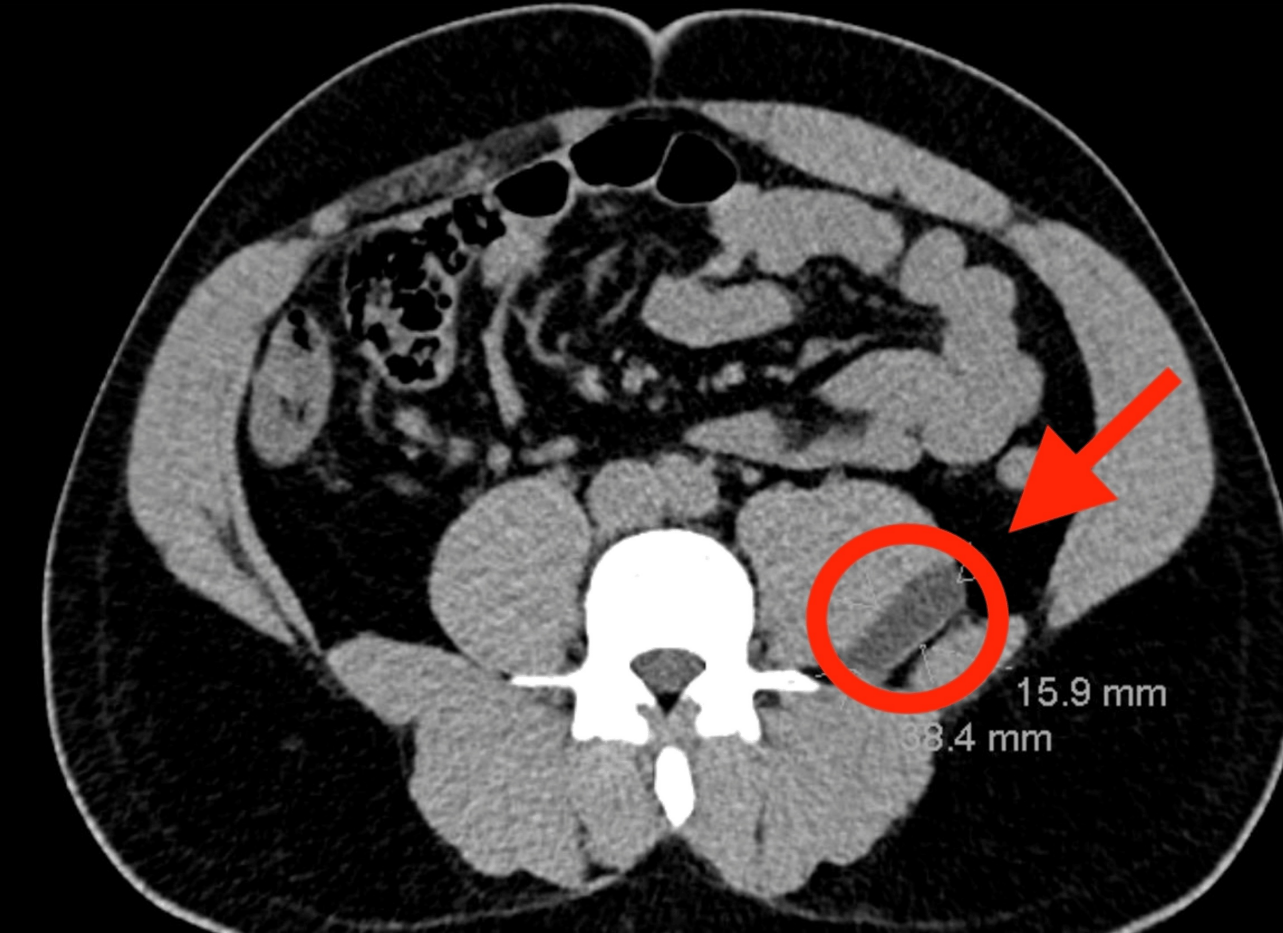
Axial CT of the patient showing psoas abscess (red arrowhead and circle) shown on the posterolateral aspect of the psoas muscle CT: computed tomography

**Figure 2:**
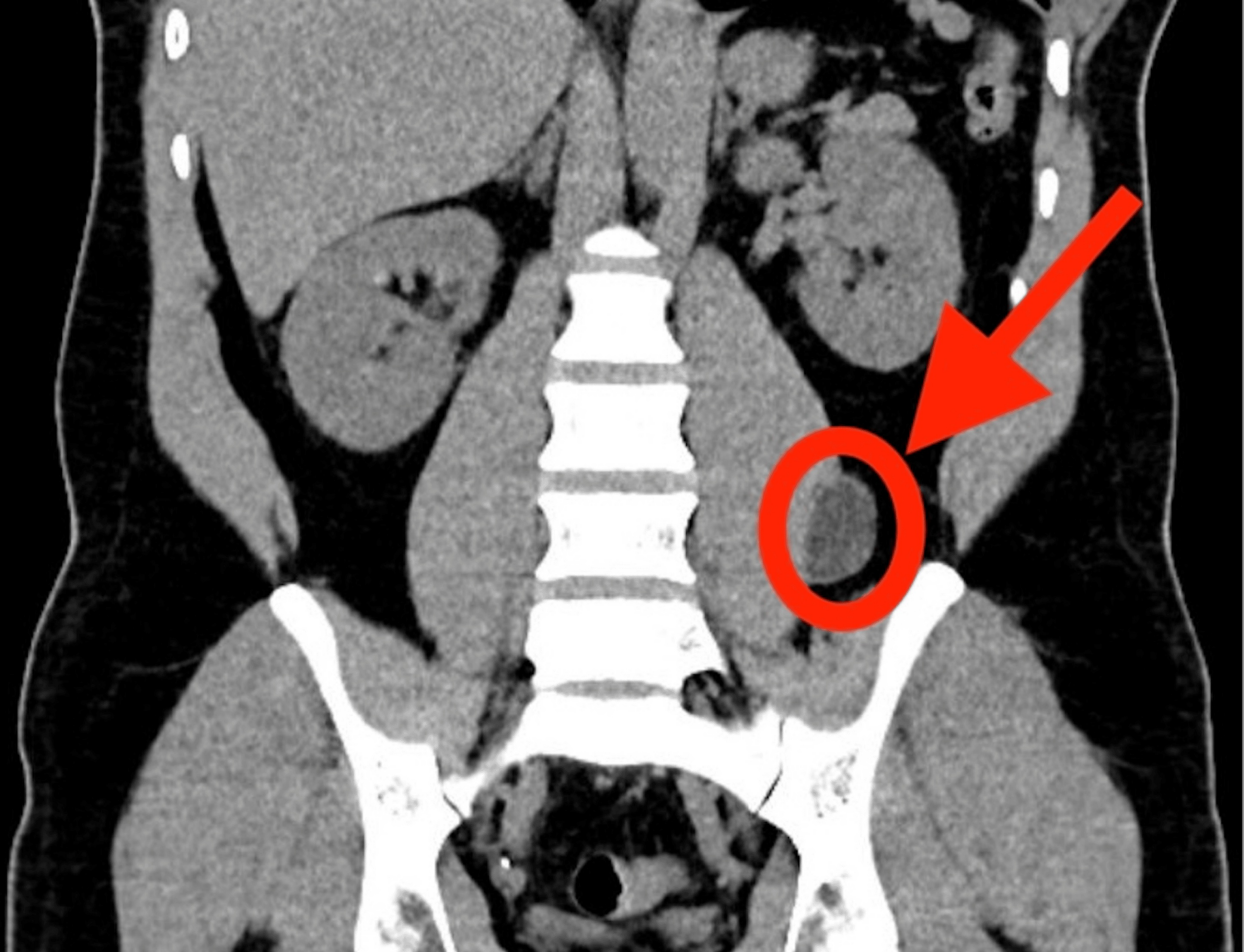
Coronal CT of the patient showing psoas abscess (red circle and arrow) shown on the lateral aspect of the left psoas muscle CT: computed tomography

However, physical examination was unremarkable and did not reveal flank or paraspinal tenderness to palpation, nor were there any limitations in hip mobility. CT of the abdomen and pelvis, shown in Figure 1 and Figure 2, revealed a 3.8 cm fluid collection adjacent to the left psoas muscle. Given the elevated WBC count and the risk for abscess, the patient was empirically treated with broad-spectrum antibiotics including ceftriaxone, Flagyl, and vancomycin.

The patient’s workup for immunologic conditions, including HIV, was negative. After admission, interventional radiology was consulted, after which they performed an image-guided drainage of the abscess. A total of 20 mL of thick yellowish fluid was drained by the interventional radiology team. Fluid cultures were unremarkable and showed no growth at 48 hours. Blood cultures were also negative, and antibiotics were subsequently discontinued. There were no complications during the admission, and the patient was later discharged with outpatient follow-up.

## Discussion

A psoas abscess is difficult to diagnose due to its variable presentation and nonspecific symptoms. Back pain was the sole complaint in our patient, and they did not exhibit any other symptoms typically seen in the classic triad. Previous literature also highlights the unreliable frequency of the classic triad in patient presentations. According to Chern et al., the classic triad was present in only approximately 30% of cases [9]. Additionally, even when diagnosed with a primary psoas abscess, this patient did not have any of the classic risk factors associated with its development. The patient's history and physical examination were otherwise unremarkable, with the only potential clue being the repetitive lifting of heavy objects at work.

Beyond the commonly recognized risk factors, a literature review revealed cases where abdominal trauma contributed to the formation of a psoas abscess. In one case, the trauma involved an 8-foot fall off a front porch; in another, it resulted from a penetrating injury to the anterior abdominal wall [10,11]. We, therefore, suggest that additional, as-yet-unrecognized etiologies for psoas abscesses may exist and warrant further investigation. However, these previously reported cases do not fully align with the presentation in our patient.

Another unique feature of this case is that, despite the presence of purulent fluid, cultures from the fluid collection showed no growth. Blood cultures were also negative, which ultimately led the medical team to discontinue antibiotic therapy. On presentation, the patient met SIRS criteria and was empirically started on antibiotics due to concern for sepsis. However, the patient's tachypnea at triage may have been attributable to pain rather than infection.

The patient’s lack of identifiable risk factors, the sterile culture results, and the unusual clinical presentation differ from what is commonly described in the literature. Therefore, we cannot determine a definitive cause for the abscess in this case. Furthermore, the absence of blood culture growth further deviates from established findings. Given the limited amount of literature on psoas abscesses, we propose that further research is needed to explore broader potential etiologies, as this case suggests a primary psoas abscess may develop through mechanisms not yet described in the literature.

## Conclusions

Psoas abscess is a diagnostically challenging condition due to its nonspecific and variable presentations. The classic triad of fever, back pain, and antalgic gait is present in only about 30% of cases, making early diagnosis difficult. Established risk factors for primary psoas abscess include diabetes mellitus, intravenous drug use, AIDS, renal failure, and immunosuppression. However, our case of a 36-year-old male with no identifiable risk factors highlights the need for clinicians to maintain a high index of suspicion for this condition. The implications of missing a psoas abscess diagnosis are significant, as delayed treatment can lead to severe complications, including sepsis and increased mortality. Therefore, emergency physicians and other healthcare providers must consider psoas abscess in the differential diagnosis of patients presenting with nonspecific symptoms such as back pain, even in the absence of traditional risk factors.

This case underscores the importance of awareness and education regarding the variable presentations of psoas abscess. By broadening the differential diagnosis and utilizing appropriate imaging modalities, clinicians can improve patient outcomes and reduce the risk of missed or delayed diagnoses.
